# A novel miR-371a-5p-mediated pathway, leading to BAG3 upregulation in cardiomyocytes in response to epinephrine, is lost in Takotsubo cardiomyopathy

**DOI:** 10.1038/cddis.2015.280

**Published:** 2015-10-29

**Authors:** M d'Avenia, R Citro, M De Marco, A Veronese, A Rosati, R Visone, S Leptidis, L Philippen, G Vitale, A Cavallo, A Silverio, C Prota, P Gravina, A De Cola, E Carletti, G Coppola, S Gallo, G Provenza, E Bossone, F Piscione, M Hahne, L J De Windt, M C Turco, V De Laurenzi

**Affiliations:** 1 Department of Pharmacy (DIFARMA), University of Salerno, Fisciano, Italy; 2 Biouniversa s.r.l., c/o University of Salerno, Fisciano, Italy; 3 Department of Biomedical Sciences and Human Oncology, University of Bari, Bari, Italy; 4 ‘Cuore' Department, University Hospital ‘San Giovanni di Dio e Ruggi d'Aragona', Salerno, Italy; 5 Dipartimento di Scienze Mediche, Orali e Biotecnologiche, CeSI, Universita' ‘G. D'Annunzio' di Chieti e Pescara, Pescara, Italy; 6 Faculty of Health, Medicine and Life Sciences, Department of Cardiology, CARIM School for Cardiovascular Diseases, Maastricht University, Maastricht, The Netherlands; 7 Department of Laboratory Medicine, ‘Tor Vergata' University, Rome, Italy; 8 Transfusional Department, University Hospital ‘San Giovanni di Dio e Ruggi d'Aragona', Salerno, Italy; 9 Division of Cardiology San Francesco d'Assisi, Hospital of Oliveto Citra, Salerno, Italy; 10 Institut de Genetique Moleculaire de Montpellier, CNRS UMR5535, Montpellier, France; 11 Department of Medicine and Surgery, University of Salerno, Fisciano, Italy

## Abstract

Molecular mechanisms protecting cardiomyocytes from stress-induced death, including tension stress, are essential for cardiac physiology and defects in these protective mechanisms can result in pathological alterations. Bcl2-associated athanogene 3 (BAG3) is expressed in cardiomyocytes and is a component of the chaperone-assisted autophagy pathway, essential for homeostasis of mechanically altered cells. *BAG3* ablation in mice results in a lethal cardiomyopathy soon after birth and mutations of this gene have been associated with different cardiomyopathies including stress-induced Takotsubo cardiomyopathy (TTC). The pathogenic mechanism leading to TTC has not been defined, but it has been suggested that the heart can be damaged by excessive epinephrine (epi) spillover in the absence of a protective mechanism. The aim of this study was to provide more evidence for a role of BAG3 in the pathogenesis of TTC. Therefore, we sequenced *BAG3* gene in 70 TTC patients and in 81 healthy donors with the absence of evaluable cardiovascular disease. Mutations and polymorphisms detected in the *BAG3* gene included a frequent nucleotide change g2252c in the BAG3 3′-untranslated region (3′-UTR) of Takotsubo patients (*P*<0.05), resulting in loss of binding of microRNA-371a-5p (miR-371a-5p) as evidenced by dual-luciferase reporter assays and argonaute RNA-induced silencing complex catalytic component 2/pull-down assays. Moreover, we describe a novel signaling pathway in cardiomyocytes that leads to BAG3 upregulation on exposure to epi through an ERK-dependent upregulation of miR-371a-5p. In conclusion, the presence of a g2252c polymorphism in the BAG3 3′-UTR determines loss of miR-371a-5p binding and results in an altered response to epi, potentially representing a new molecular mechanism that contributes to TTC pathogenesis.

Takotsubo cardiomyopathy (TTC), also known as stress cardiomyopathy or ‘broken heart syndrome', is characterized by transient and reversible myocardial stunning leading to systolic left ventricular (LV) apical ballooning in the absence of obstructive coronary artery disease. In general, it occurs in postmenopausal women and is triggered by emotional or physical stress.^1^ The long-term prognosis of TTC patients is favorable due to spontaneous recovery of myocardial function. Up to date the pathogenic mechanism leading to this disease has not been defined,^1^ although it has been suggested that the heart muscle can be damaged by excessive epinephrine (epi) release,^2^ suggesting that a protective mechanism might fail in these subjects.

Bcl2-associated athanogene 3 (BAG3) is a member of the BAG family of co-chaperones that interacts with the ATPase domain of the heat shock protein 70.^3^ Under physiological conditions, BAG3 expression is restricted to few cell types including cardiomyocytes.^4, 5, 6^ Its expression can be induced by a variety of stressors and it is thought to contribute to stress resistance.^7, 8, 9^ The presence of proteins that protect cardiomyocytes from stress-induced apoptosis has a crucial role in the pathophysiology of the myocardium. Indeed, cardiomyocytes are intrinsically resistant to apoptosis, in part due to high levels of endogenous caspase inhibitors,^10^ anti-apoptotic Bcl-2 proteins and the pro-survival kinase Akt.^11^ In line, we found that BAG3 levels increase during myoblast differentiation, suggesting that its biological role is relevant for differentiated myocytes.^5^ This is in agreement with the observation that *Bag3* deletion causes a lethal cardiomyopathy not in the embryos but in postnatal *Bag3*-deficient mice.^12^ Moreover, it was found that *Bag3* silencing results in highly reduced myogenin levels.^5^ These findings indicate an involvement of BAG3 protein in late heart development and are in keeping with the role of BAG3 in the survival and myofibrillar integrity in cardiomyocytes. Several reports associate *BAG3* mutations with myopathy. Selcen *et al.*^13^ described a mutated form of BAG3, that is, heterozygous Pro209Leu, which caused a severe and progressive muscle weakness in childhood cardiomyopathy. In addition, non-synonymous *BAG3* single-nucleotide polymorphisms (SNPs) or other truncated BAG3 forms correlate with familiar dilated cardiomyopathy (DCM)^14^ and stress cardiomyopathy also known as TTC.^15^ Finally, two heterozygous *BAG3* gene mutations, which cause abnormal *Z*-disc assembly and increased sensitivity to apoptosis in cultured cardiomyocytes, were identified in patients with familial DCM.^16^ These findings, together with our observation that BAG3 protein is released from stressed cardiomyocytes and can be detected in sera of patients with chronic heart failure (HF),^6^ strongly suggest that BAG3 has a role in the regulation of cardiomyocyte survival under stress conditions.

In this study we describe a novel posttranscriptional pathway leading to BAG3 induction on epi treatment. MicroRNAs (miRNAs) are small non-coding RNAs that modulate gene expression by incomplete base pairing to their cognate target messenger RNAs (mRNAs), resulting usually in translational repression.^17^ miRNAs have a critical role in the normal maintenance of fundamental cellular processes and their deregulation could affect a large number of molecular pathway in human diseases.^18, 19, 20, 21^ Among these, the cardiac pathologies are correlated to miRNAs^22, 23, 24^ and, in recent times, changes in levels of specific circulating miRNA have been reported in TTC patients.^25^ There are only a few studies that show that miRNAs can also enhance mRNAs translation.^26, 27^ Here we describe a posttranscriptional pathway involving the binding of an miRNA to the 3′-untranslated region (3′-UTR) of the *BAG3* gene, resulting in increased BAG3 expression. We find that epi induces miR-371a-5p, resulting in increased BAG3 protein expression. We also show that one nucleotide variant in the 3′-UTR of the *BAG3* gene, frequently found in TTC patients, results in alteration of this posttranscriptional pathway.

## Results

### *BAG3* gene is frequently mutated in Takotsubo patients

We have recently reported two TTC-related missense mutations in the *BAG3* coding region in a cohort of 29 patients^15^ and extended our study by screening a total of 70 women TTC patients. As a control group, we used a group of female donors over the age of 50 years, to reduce the possibility that control donors will develop the disease in the future, as the reported mean age of onset ranges from 58 to 75 years in the different reports.^28^ We sequenced exons 2–4 of the coding sequence and the entire 3′-UTR of *BAG3*. We were not able to consistently amplify exon 1 (as reported before^29^) and therefore have not included data on this exon. Tables reporting all sequence mutations found are presented in Supplementary Tables S1 and S2.

TTC patients had a higher frequency of mutations in the *BAG3* gene in comparison with healthy donors. In fact, Table 1 shows that only 27.1% of the TTC patients analysed had no mutation in the *BAG3* sequence as compared with 53.1% of healthy donors. Moreover, 21.4% of TTC patients and only 12.3% of the donors showed a homozygous nucleotide change in the *BAG3* sequence. Furthermore, 47.1% of TTC patients but only 29.6% of the controls had more than one *BAG3* mutation and were therefore potentially carrying two altered alleles. We cannot exclude that sequencing the remaining part of the coding sequence and the 5′-UTR of *BAG3* gene would not result in the discovery of additional mutations in the TTC cohort, thus improving the significance of genetic analysis.

Among the genomic variants identified, a particularly frequent mutation in the 3′-UTR (g2252c-SNP rs8946) was identified (Supplementary Table S3). Indeed, 62.8% of TTC patients carried this nucleotide variant, of which 12.8% were homozygous for the g2252c variant. In contrast, this mutation was present in only 45.6% of the samples of the control group and only 7.4% were homozygous (the two-tailed *P*-value obtained by Fisher's exact test on the 2 × 2 contingency table equals 0.04). Moreover, despite the fact that control samples were all from female donors over 50 years of age, it is possible that some of the controls carrying this mutation, in particular the homozygous cases, will develop the symptoms if exposed to a strong triggering event. We therefore executed a series of experiments to determine what molecular pathway the g2252c variant may potentially alter in human cardiac myocytes.

### miR-371-5p increases BAG3 protein levels

To investigate whether the nucleotide change g2252c may affect BAG3 expression levels by altering the binding of regulatory miRNAs, we performed an *in-silico* analysis of the sequence and identified a number of potential miRNAs that were predicted to bind the sequence containing this nucleotide change (Supplementary Table S4). Among those, miR-371a-5p (miR-371a) (MI0000779) showed the highest predictive score and was therefore chosen for further investigations. TargetScan algorithm identified miR-371a-5p-binding region on the BAG3 3′-UTR as a ‘poorly conserved site for miRNA families conserved only among mammals or vertebrates'. Moreover, a sequence analysis among species, performed by mVISTA alignment tool,^30^ highlights that only humans have the correct binding site on BAG3 3′-UTR for hsa-miR-371a-5p, which is missing in mouse, rat, pig, chimpanzee and gorilla (Supplementary Figure S1).

**Figure 1:**
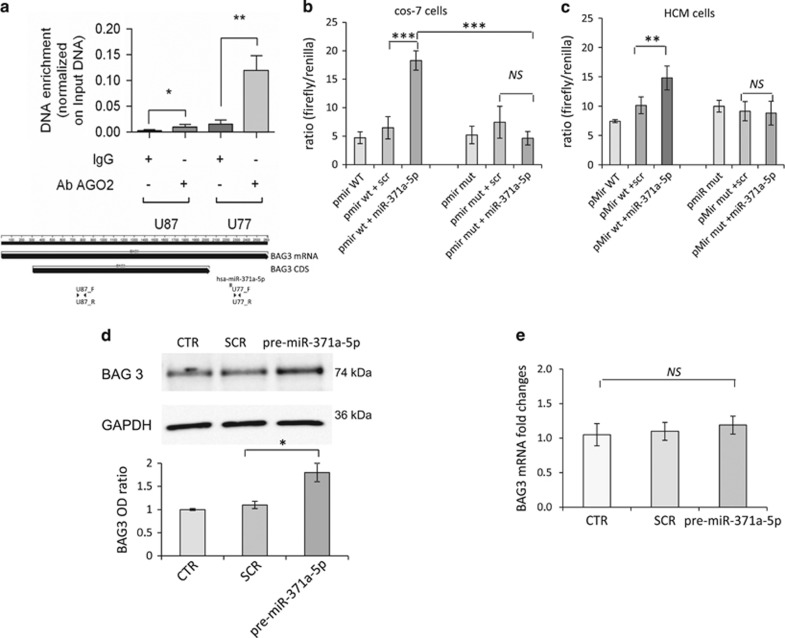
miR-371a-5p regulates expression of BAG3. To test whether the predicted miRNAs could bind BAG3 target, both Ago2/pull-down assay and pMir dual-luciferase assay were performed. mRNA from HEK293 cells was IP with the anti-AGO2 antibody and BAG3 mRNA was quantized by quantitative RT-PCR (**a**). Data were normalized on the pri-miRNA 15a/16-1 expression of the RNA, not IP (INPUT). In the lower part of **a** there is also a schematic representation of the *BAG3* gene. BAG3 mRNA and BAG3 coding sequence (black arrows), hsa-miR-371-5p targeted sequence (red bar) and fragments used for the qRT-PCR in the RIP analysis are shown. Cos-7 (**b**) and HCMa cells (**c**) were co-transfected with the pre-miR-371a-5p (0.1 nmol/l) (miR-371a-5p) or a SCR control (scr) and a firefly *luciferase* pMir reporter plasmid containing downstream the *luciferase* gene, the wt (pmir WT) or mutated (g2252c) (pmir mut) *BAG3* 3′-UTR sequence. Luciferase activity differences among samples were assayed 24 h after transfection and *Renilla* luciferase was co-transfected as a control. Data represent the mean of six experimental replicates; *P*-value *versus* SCR and the mutated sequence are shown. HCMa cells were transfected with pre-miR-371a-5p (2 nmol/l) (pre-miR-371a-5p) or SCR sequences (SCR) as a negative control, and BAG3 protein (**d**) and mRNA levels (**e**) were evaluated after 24 h. (**d**) Densitometric analysis of five blots, normalized to the corresponding GAPDH band intensities, is shown in **d** (lower panel). BAG3 mRNA levels, evaluated by real-time RT-PCR, were compared in three independent experiments using the relative ct method: the amount of target, normalized to the amount of endogenous control (GAPDH), relative to the control sample, is given by 2^–ΔΔCt^. Significance calculated by *t*-test: ***P<0.0001, **P<0.005 and *P<0.01; NS, not significant

By immunoprecipitating the argonaute RNA-induced silencing complex (RISC) catalytic component 2 (AGO2) protein in HEK293 cells using RNA-binding protein immunoprecipitation or RNA-binding protein immunoprecipitation (RIP) assay, we confirmed that the RISC complex binds to the BAG3 3′-UTR (Figure 1a). In addition, the starBase database (http://starbase.sysu.edu.cn/index.php) that harbors the interaction map from Argonaute CLIP-seq data^31, 32^ also demonstrated the specific binding of the miR-371a-5p to the BAG3 3′-UTR. To further experimentally validate whether miR-371a-5p directly binds to the 3′-UTR of BAG3 and evaluate whether this binding is affected by the g2252c nucleotide change, we performed dual-luciferase reporter assays using pMIR-reporters with either the wild-type (wt) or polymorphic putative miRNA target sites cloned downstream of the firefly luciferase gene (*luc2*). In this experiment, firefly luciferase was used as the primary reporter to monitor gene regulation and *Renilla* luciferase (hR*luc-neo*) acted as a control reporter for signal normalization. Cos-7 cells and a primary adult human cardiomyocyte adult (HCMa) cell line were transiently transfected with precursor molecules for the predicted miRNAs or a scrambled (SCR) control together with the pMIR-reporter plasmids, harboring either the wt BAG3 3′-UTR or the BAG3 3′-UTR carrying the homozygous g2252c mutation. Our results demonstrate that miR-371a-5p increased luciferase activity in the reporter harboring the wt BAG3 3′-UTR but failed to increase reporter activity in the reporter with the mutated BAG3 3′-UTR (Figures 1b and c). Consistent with this finding, precursor transfection of miR-371a-5p resulted in increased BAG3 protein levels in HCMa cells (Figure 1d). Notably, no changes in BAG3 mRNA levels were observed (Figure 1e), suggesting that miR-371a-5p acted by influencing BAG3 translation rather than BAG3 mRNA stability. Taken together, these data demonstrate that miR-371a-5p enhance BAG3 protein expression, and that the interaction of miR-371a-5p with human BAG3 3′-UTR critically depends on the presence of the g2252 variant.

### Epi upregulates BAG3 levels through miR-371a-5p regulation

**Figure 2:**
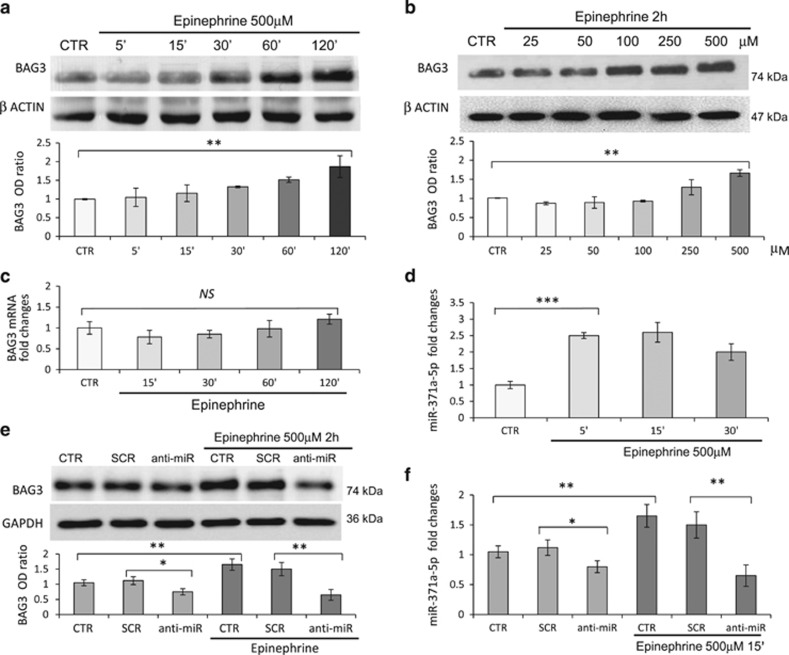
Epi induces BAG3 overexpression through miR-371a-5p. HCMa cells were treated with epi HCl (at the indicated time points (**a**) or concentrations (**b**)) and collected. WB analysis of BAG3 protein is shown (**a** and **b**). One of six independent experiments is shown and densitometric analysis of experimental replicates, normalized to the corresponding *β*-actin band intensities, are shown in the lower part of **a** and **b**. (**c**) BAG3 mRNA levels after epi kinetics, evaluated by real-time RT-PCR, normalized to the amount of endogenous control (GAPDH) and relative to the control sample, are given by 2^–ΔΔCt^. (**d**) miR-371a-5p levels were detected by real-time RT-PCR: transcript quantities were compared using the relative Ct method, where the amount of the target, normalized to the amount of endogenous control (RNU6) and relative to the control sample is given by 2^–ΔΔCt^. To silence the miRNA activity and generate a transient miRNA-371a-5p knockout model, cardiomyocytes were transfected with the anti-miR-371-5p (anti-miR) or SCR sequences as a negative control (10 nmol/l) and 24 h later stimulated with epi (500 *μ*mol/l) for 2 h or 15 min. Cells were subsequently analysed by WB, to evaluate BAG3 protein levels (**e**), and by real-time RT-PCR, to evaluate miR-371a-5p levels (**f**), as described above. Data are representative of three independent experiments with similar results and WB densitometric analysis of experimental replicates, normalized to the corresponding GAPDH band intensities, are shown in the lower part of **e**. Significance *versus* the SCR or the control samples were calculated by *t*-test: ***P<0.0005, **P<0.005 and *P<0.01; NS, not significant

Exposure to high levels of epi, either as a consequence of strong emotional stress or as a consequence of administration of catecholamines or the presence of inhibitors of serotonin/norephinephrine re-uptake inhibitors, has been implicated in TTC.^33, 34^ We therefore investigated whether epi could modulate BAG3 protein expression in adult human cardiomyocytes and whether miR-371a-5p could participate in this regulation. In line with our expectations, epi treatment of HCMa cells resulted in a dose- and time-dependent increase of BAG3 protein levels (Figures 2a and b). Under these experimental conditions, epi treatment resulted in activation of the adrenergic response as shown by intracellular cyclic AMP (cAMP) increase (Supplementary Figure S2) but not yet in cell death (Supplementary Figure S3). By using selective adrenergic receptor inhibitors we demonstrated that epi-dependent BAG3 regulation critically depended on the stimulation of *α*1 receptor stimulation (Supplementary Figure S4). Interestingly, BAG3 mRNA levels remained constant in response to epi stimulation, indicating a posttranscriptional regulatory mechanism in the regulation of BAG3 protein expression (Figure 2c). Moreover, treatment with epi also resulted in higher expression of miR-371-5p, indicating that this miRNA may have a role in the observed BAG3 induction in response to epi (Figure 2d). To confirm that the induction of BAG3 by epi indeed depends on the regulation of miR-371-5p, we transfected HCMa cells with anti-miR-371-5p to silence endogenous miR-371-5p. Transfection with anti-miR-371-5p, but not with a SCR control, resulted in a modest reduction of BAG3 basal protein levels, but completely abrogated the induction of BAG3 protein by epi (Figure 2e). Real-time reverse-transcription PCR (RT-PCR) confirmed that the anti-miR molecule was capable of reducing miR-371a-5p expression even in the presence of epi (Figure 2f).

**Figure 3:**
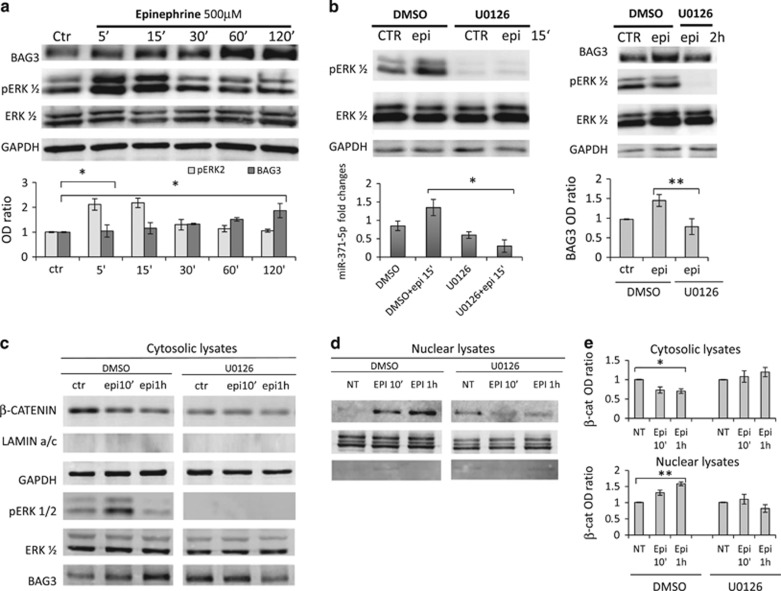
Epi induces ERK phosphorylation, *β*-catenin translocation and miR-371a-5p transcription, leading to BAG3 upregulation. (**a**) HCMa cells were treated with epi HCl (500 *μ*mol/l) and collected at the indicated time points, and cell lysates were analysed by WB with antibodies for BAG3, phosphorylated ERK (pERK1/2) and total ERK (ERK1/2). GAPDH was used as loading control; BAG3 and pERK densitometric analysis, normalized to the corresponding GAPDH or total ERK1/2 band intensities, are shown in the lower part of **a**. (**b**) Cells were pre-treated for 30 min with phospho-ERK inhibitor U0126 (10 *μ*mol/l in dimethyl sulfoxide (DMSO)), or solvent alone, and for an additional 15 min (left) or 2 h (right) with epi HCl (epi) (500 *μ*mol/l); cell extracts were analysed by WB for BAG3, phosphorylated ERK (pERK1/2) and total ERK (ERK1/2). GAPDH was used as loading control (upper panels). The left lower panel shows miR-371a-5p expression levels, analysed by real-time RT-PCR. Transcript quantities were compared using the relative Ct method as described before. Cytosolic (**c**) and nuclear (**d**) fractions of HCMa cells pretreated with U0126 as described before and then treated with epi at the indicated times were analysed separately by WB for BAG3, phosphorylated ERK (pERK1/2), total ERK (ERK1/2) and *β*-catenin. GAPDH and LAMIN A/C were used as loading control in cytosolic and nuclear fraction. Images shown are representative of three independent experiments. (**e**) Densitometric analysis of *β*-catenin signals, normalized to the corresponding GAPDH (cytosol) or LAMIN A/C (nucleus). Significance calculated by *t*-test: *P<0.01 and **P<0.001

We next investigated the molecular pathway leading to miR-371a-5p induction by epi. Interestingly, treatment of HCMa cells with epi also resulted in ERK phosphorylation at about the same time when the increase of miR-371a-5p is observed (Figures 3a and b, lower left panel) and treatment with U0126, an inhibitor of ERK phosphorylation, blocked epi-induced upregulation of miR-371a-5p and BAG3 levels (Figure 3b, respectively, left and right side).

**Figure 4:**
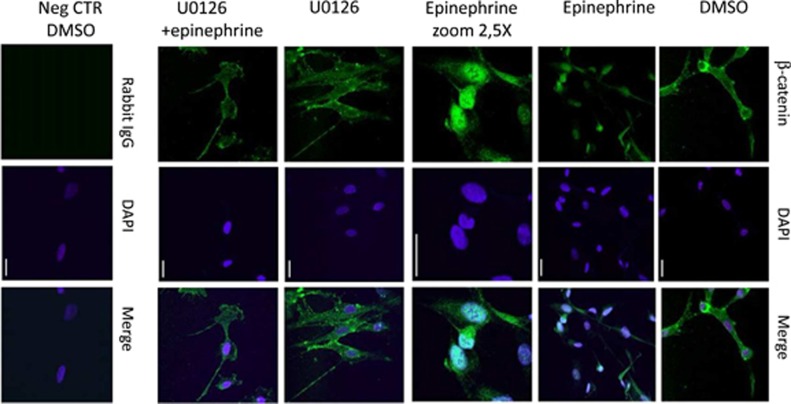
Epi induces *β*-catenin nuclear translocation. HCMa were pretreated with phospho-ERK inhibitor U0126 (10 *μ*mol/l in dimethyl sulfoxide (DMSO)), or solvent alone, and then stimulated with epi 500 *μ*mol/l for 10 min. For indirect immunofluorescence, cells were stained for *β*-catenin detection with an anti-rabbit FITC-488-conjugated Ab. Negative control rabbit IgG were used in place of a primary rabbit polyclonal antibody to evaluate nonspecific staining. Hoechst 33342 was used for nuclear staining (DAPI). Samples were analyzed by using a confocal microscope, (objective × 63; bars=20 *μ*m)

**Figure 5:**
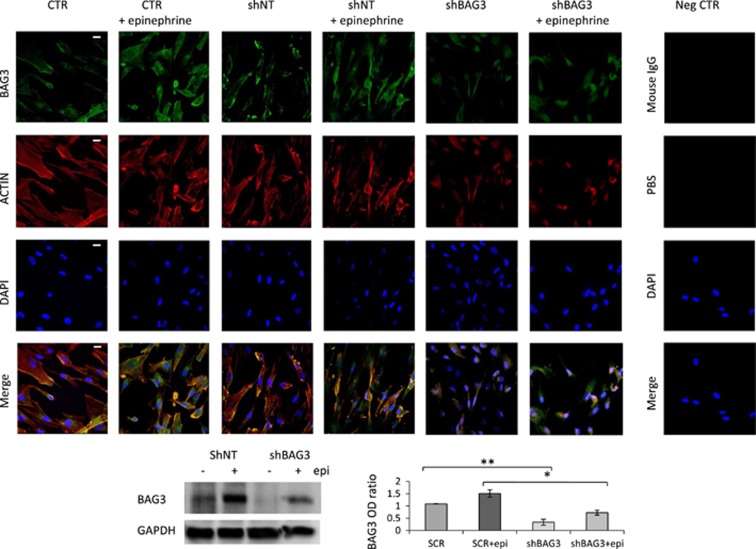
BAG3 functional role in epi-stimulated cardiomyocytes: epi induces an increase of BAG3 expression and supports BAG3-F-actin co-localization; moreover, BAG3 expression is essential for F-actin fiber structure. HCMa were seeded 60 × 10^3^ cells/well (6-well plates) and 4 h later were transfected with a human BAG3 shRNA or a hu-shNT no-target vector as negative control (0.5*μ*g/well), to silence the *BAG3* gene. After 72 h, cells stimulated with epi 500 *μ*mol/l for 2 h. For indirect immunofluorescence, cells were stained for BAG3 detection with an anti-mouse FITC-488-conjugated Ab. Negative control mouse IgG were used in place of a primary mouse mAb, to evaluate nonspecific staining. To stain F-actin filaments, cells were incubated with TRITC-conjugated Phalloidin. Hoechst 33342 was used for nuclear staining (DAPI). Samples were analyzed by using a confocal microscope (objective × 60; bars=20 *μ*m). Human BAG3 shRNA efficiency in silencing BAG3 was assayed also by WB. Images shown are representative of three independent experiments and densitometric analysis of BAG3 WB signals, normalized to GAPDH, are shown. epi, epinephrine; shNT, short hairpin RNA no target; shBAG3, human BAG3 short hairpin RNA. Significance calculated by *t*-test: *P<0.01 and **P<0.001

It has been shown that the promoter region of the miR-371-373 cluster contains TCF/LEF1-binding elements and that *β*-catenin is required for Wnt-dependent induction of the miR-371-373 cluster.^35^ We therefore tested the possibility that epi treatment resulted in translocation of *β*-catenin to the nucleus. Indeed, as demonstrated in Figures 3c–e and Figure 4, treatment with epi resulted in a rapid translocation of *β*-catenin to the nucleus. This was dependent on ERK phosphorylation, as U0126 efficiently blocked *β*-catenin nuclear translocation (Figures 3c–e and Figure 4), as well as the increase in BAG3 expression (Figure 3b right panel and Figure 3c). Finally, we also demonstrated that BAG3 co-localizes with F-actin filaments in cardiomyocytes and is essential for their correct assembly. In line, silencing of BAG3 in HCMa resulted in altered F-actin filament structure as well as a reduction of actin levels (Figure 5).

Taken together, these data demonstrate that in human cardiomyocytes, epi induces ERK phosphorylation-dependent *β*-catenin nuclear translocation, resulting in increased miR-371-5p expression,^35^ which in turn influences BAG3 protein expression level. Correct BAG3 induction appears to be a required component for correct sarcomere assembly in cardiomyocytes. Intriguingly, this posttranscriptional pathway regulating BAG3 protein expression is altered by the presence of the g2252c variant in the 3′-UTR of human BAG3, which is frequently present in a cohort of TTC patients, suggesting that epi-induced miR-371-5p and BAG3 may potentially represent new molecular components in the disease pathogenesis of TTC.

## Discussion

**Figure 6:**
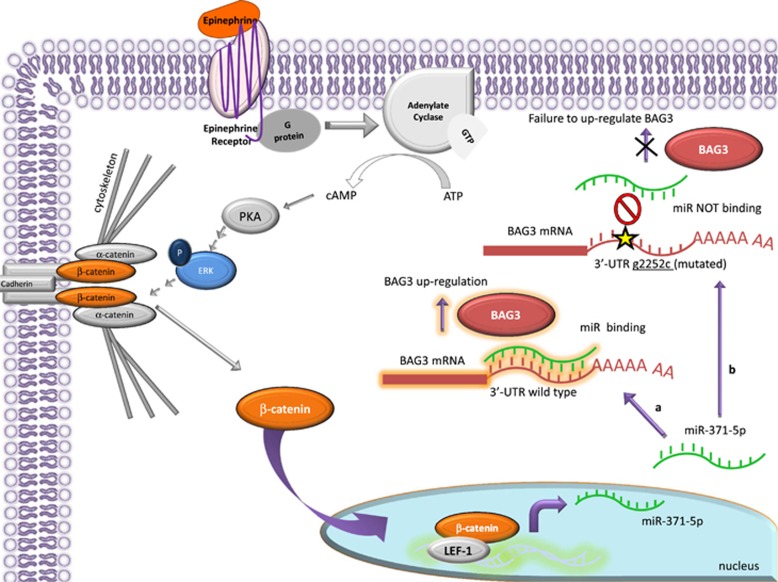
Epi regulatory pathway on BAG3 expression. Epi in cardiomyocytes induces, possibly through PKA,^42^ ERK phosphorylation. Phosphorylated ERK was also shown to phosphorylate CK2*α*, which then phosphorylates *α*-catenin, abrogating its inhibitory effect on *β*-catenin as previously described.^43^ *β*-Catenin is indeed free to translocate into the nucleus where it acts as transcription factor, together with LEF-1, enhancing mir-371–373 cluster transcription.^35^ Mature miR-371a-5p binds the wt BAG3 3′-UTR enhancing protein translation (**a**). When the homozygous g2252c mutation occurs, miR-371a-5p can no longer bind to the 3′-UTR of BAG3 and no increase of BAG3 is obtained in response to epi (**b**)

Our data provide evidence for a new posttranscriptional epi-induced mechanism involving miR-371-5p binding to BAG3 3′-UTR that may fail in subjects carrying *BAG3* genomic variants. TTC, or the ‘broken heart syndrome', is characterized by transient and reversible myocardial stunning leading to systolic LV apical ballooning, is more prevalent in post-menopausal women and is triggered by emotional or physical stress by excessive epi release.^1, 2^ In our proposed mechanism, epi increases BAG3 protein levels through direct miR-371a-5p binding to the 3′-UTR region of BAG3 as demonstrated by the dual-luciferase assay. Interestingly, SNP rs8946 lies in this region and our data suggest that this nucleotide change, which is more frequently present in TTC patients, prevents or destabilizes the binding of miR-371a-5p on BAG3 3′-UTR. In more detail, we show that in cardiomyocytes epi triggers ERK phosphorylation that in turn results in *β*-catenin translocation to the nucleus. It has previously been shown that nuclear *β*-catenin cooperates with the transcription factor LEF-1, to promote expression of miR-371-5p^35^ (Figure 6). Interestingly, in the mechanism we describe, the direct binding of miR-371a-5p to the 3′-UTR of BAG3 results in increased expression of BAG3 protein presumably through translational regulation, which requires further analyses in our future studies. Although miRNAs have primarily been implicated in posttranscriptional repression of target protein, accumulating evidence indicate that miRNAs and their associated protein complexes can also stimulate gene expression at the posttranscriptional level by a variety of direct and indirect mechanisms.^26, 27, 36, 37^

Based on the current knowledge on the functional role of BAG3 in cardiomyocytes, it is reasonable to propose that the loss of BAG3 regulation may sensitize cardiomyocytes to stress conditions. In line, it has recently been shown that BAG3 is essential for homeostasis of mechanically stressed cells,^38, 39^ being an important component of the chaperone-assisted autophagy (CASA) pathway leading to selective lysosomal degradation of unfolded proteins. In muscle cells, the CASA machinery is located at the *Z*-disk and appears to be essential for disposal of unfolded mechano-sensors and cytoskeleton proteins resulting from mechanical tension. Impairment of the CASA machinery results in *z*-disk disruption in contracting muscles.^38, 39^ Moreover, it has been demonstrated that mutations of *BAG3* result in abnormal muscle function and are responsible for both muscle dystrophies and cardiomyopathies, whereas reduced BAG3 levels contribute to disease onset in non-familiar cases of dilated cardiomyopathies.^40^ Moreover, BAG3 depletion causes a lethal cardiomyopathy not in the embryos but in postnatal bag3-deficient mice.^12^ In line with these reports, we found that silencing BAG3 in primary adult human cardiomyocytes results in a dramatic alteration of F-actin filaments. Our studies may suggest that basal levels of BAG3 in TTC patients are sufficient to prevent muscle cell alterations, and that the g2252c mutation in the 3′-UTR of BAG3 could potentially result in the loss of the ability to upregulate this protein on exposure to high levels of epi. The absence of BAG3 regulation might be generally well tolerated; however, under particularly stressful conditions, failure to upregulate BAG3 to a sufficient level might result in cardiac damage and an overt clinical phenotype.

In conclusion, we identified a novel signaling pathway leading to BAG3 upregulation in response to epi in human cardiomyocytes and this pathway may potentially be less active in subjects carrying the g2252c nucleotide change in homozygosis and impaired in those carrying it in heterozygosis. It is possible that the few control cases showing this mutation in homozygosis might develop the disease later in life only if subjected to strong emotional stress or as a consequence of iatrogenic exposure to catecholamines. In subjects carrying the *BAG3* g2252c polymorphism in heterozygosis, it is possible that additional variants that impair BAG3 function or regulation occur in the other allele and contribute to the disrupted function of BAG3. In conclusion, the significantly increased frequency of the g2252c nucleotide change in patients with Takotsubo provides circumstantial evidence that the g2252c variant may predispose cardiomyocytes to epi-induced damage and could potentially have a functional role in the pathogenesis of the disease.

## Materials and Methods

### Patients and donors

All patients were enrolled according to the Mayo Clinic diagnostic criteria of TTC as follows: transient akinesia or dyskinesia of LV apical and/or midventricular segments; no angiographic evidence of ≥50% coronary artery stenosis or plaque rupture, or intracoronary thrombus formation; new electrocardiogram (ECG) abnormalities (dynamic ST–T changes or T-wave inversion); and the absence of intracranial bleeding, pheochromocytoma and myocarditis.

Blood samples were collected from the University Hospital ‘San Giovanni di Dio e Ruggi d'Aragona', Salerno, Italy, in a period of 3 years from January 2011 to December 2013, and from San Luca Hospital, Vallo della Lucania, Salerno, Italy, in a period of 3 years from January 2007 to December 2009. All participants provided informed written consent, the study was approved by the local ethics committee and conforms to the principles of the Declaration of Helsinki.

Takotsubo patient cohort included 70 women (mean age 64.3±12; range from 35 to 82 years). In the overall population, among typical cardiovascular risk factors, hypertension was prevalent (55%) compared with diabetes, hypercholesterolemia and smoking. The majority of the patients (82%) were in menopausal state. An acute trigger event, preceding the onset of TTC, was reported in 52 patients (emotional and physical trigger in 41 and 11 patients, respectively). Chest pain was the prevalent symptom at the time of admission in 44 patients (62%). At ECG presentation, ST elevation was more frequent (56%) compared with non ST elevation (18%) and T-wave inversion (26%). There was a striking prevalence (81%) of apical ballooning. Variant forms were detected in 13 patients (11 mid-ventricular and 2 basal ballooning). LV systolic function was markedly reduced 34±8%. As reported in larger series of TTC patients, acute complications occurred in 28% of patients. HF was the most common complication detected in 14 patients, whereas cardiogenic shock was diagnosed in 6 patients.

As a control group, we sequenced DNA samples from 81 healthy female donors, over the age of 50 years, with the absence of evaluable cardiovascular disease at the moment of blood collection. Control samples were collected from the Immune Transfusional Department of the University Hospital ‘San Giovanni di Dio e Ruggi d'Aragona'.

### Sequence analysis

*BAG3*-specific primers were designed to amplify by PCR exon 2 (Fw5′-AGGAGGGTTCACTTCCCAGT-3′, Rw5′-CCCACTGAAGAACAGCCCTA-3′), exon 3 (Fw5′-TGCCCTCTACCCTGTGTCTC-3′, Rw 5′-CACCCCTGGAGACATACCAC-3′), exon 4 (Ist part) (Fw5′-TTCCCAGCCTGAAAACAAAC-3′, Rw5′-CTGGACTTGACCTGGGACAT-3′) and exon 4 (IInd part) (Fw5′-GAGGGACGAGCCGATGTGCG-3′, Rw5′-AGGTGGTGGGGGTGCCCAAG-3′), by using as template 50 ng of genomic DNA (gDNA), purified from whole fresh blood (200 *μ*l), (DNeasy Blood and Tissue Kit; Qiagen, Valencia, CA, USA) collected from patients and healthy donors. PCR products (20–100 ng) were sequenced using BigDYE v3.1 (1 *μ*l; Applied Biosystems, Foster City, CA, USA), SBDD buffer (1.9 *μ*l; Applied Biosystems), Fw or Rw primer (5 pmol) and water to reach the final volume of 10 *μ*l, by following the supplier instructions for cycling conditions. The reaction volumes were purified by precipitation with ethanol and NaAc (50 mmol/l), and shipped to the sequencing facility.

### Bioinformatic analysis

Sequencing data were analyzed and aligned using ClustalW software (available at www.clustal.org, UCD Dublin, Ireland). We assessed miRNA-binding potential to the BAG3 3′-UTR at position 2252 (±10 bp) using open computational software tools developed by the Segal Lab of Computational Biology (http://genie.weizmann.ac.il/), miRBase, miRWalk and TargetScan. Sequence conservation analysis among species was performed using the mVISTA tool.^30^

### Cell cultures, treatments and transfections

HCMa were purchased both from Sciencell Research Laboratories (San Diego, CA, USA) and Promo Cell GmbH (Heidelberg, Germany), and grown respectively in cardiac myocyte medium supplemented with fetal bovine serum (FBS) (5%), cardiac myocyte growth factors (1%), penicillin/streptomycin solution (1%) (Sciencell Research Laboratories) or myocyte growth medium supplemented with fetal calf serum (5% V/V), human epidermal growth factor 0.5 ng/ml, basic fibroblast growth factor 2 ng/ml, insulin 5 *μ*g/ml (Promo Cell GmbH) (Supplementary Figure S5).

All experiments were performed on low-passage cell cultures. Cos-7 cells (ECACC 87021302) were cultured in DMEM supplemented with FBS (10%) and penicillin/streptomycin (1%) solution in a 5% CO_2_ incubator at 37 °C. Transfections were performed using serum-free media. Cardiomyocytes were seeded 50 000 cells/well (12-well plate for protein expression analysis) or 100 000 cells/well (6-well plate for mRNA and miRNA expression analysis) and transfected with precursor (2, 5 and 10 nmol/l) or inhibitors (1, 10 and 100 nmol/l) of miR-371a-5p or SCR sequences as a negative control (Ambion, Foster City, CA, USA), by using Oligofectamin (Invitrogen). HCMa cells were stimulated with epi HCl (25, 50, 100, 250 and 500 *μ*mol/l for 5, 10, 15, 30, 60 and 120 min) (Sigma Aldrich, St. Louis, MO, USA) and cells were then collected in the Cell Disruption Buffer provided by the miRVana Paris Kit (Ambion) at the indicated time points. pERK inhibition was obtained by pretreating cells with the pERK inhibitor U0126 (10 *μ*mol/l) (Calbiochem, San Diego, CA, USA) for 20 min, then epi was added (500 *μ*mol/l). To assay which epi receptor is involved in the signaling, cells were pretreated for 30 min with *α*1 antagonist Prazosin (5 *μ*M), *β*2 antagonist ICI 118–551 (1 *μ*M), *β*1 antagonist Atenolol (10 *μ*M) and then stimulated for 2 h with epi HCl (500 *μ*M). Dimethyl sulfoxide (Sigma Aldrich) was used as a solvent control for U0126 and epi receptor antagonists, and its concentrations never exceeded 0.1% v/v. To silence BAG3 expression, HCM cells were seeded 60 000 cells/well (6-well plates) and 4 h later transfected by using 2 *μ*l of X-treme Gene 9 reagent (Roche, Indianapolis, IN, USA) with 0.5 *μ*g/well of BAG3-human 29mer shRNA retroviral construct (gene ID=9531; Origene Cat.Nr.TR314524) or HuSH 29-mer shRNA non-effective expression (No target; Origene Cat.Nr.TR30003) for 72 h.

### RNA-binding protein immunoprecipitation assay

RIP assay was performed with Magna RIP kit (Millipore, Billerica, MA, USA) according to the manufacturer's instruction. HEK293 cells were lysed and the RNA-associated proteins were immunoprecipitated (IP) with anti-AGO2 Antibody (Millipore, 03-110). The precipitated RNA was retro-transcribed and measured by quantitative real-time PCR (Roche, Universal Probe Library System) by normalization to the non-IP control (Input). Primers were designed using the Universal Probe Library Assay Design Center (Roche). Primers and probes sequences were as follows: U87_BAG3_741F 5′-TCAGCCAGATAAACAGTGTGGA-3′, U87_BAG3_826R 5′-GAGACTGGGACCGCTCAG-3′ with the UPL#87 (Roche) and U77_BAG3_2298F 5′-TCTGCAGCCCTGTCTACTTG-3′, U77_BAG3_2338R 5′-AGACAGTGCACAACCACAGC-3′ with the UPL#77 (Roche).

### Plasmids and luciferase assay

BAG3 (NM_004281.3) 3′-UTR, 504-bp long, encompassing the predicted miRNA targeting sequence, was amplified by PCR by using the modified primer pair FW5′-TCATGTATAGAGCT|CCTCTGCCCTGTAAAAATCAGA-3′(*Sac*I) and RW5′-TCATGTATAA|AGCTTAAAATGTAGCATTAAAGTCATCCAA-3′(*Hind*III), and gDNA template isolated from a patient carrying the SNP rs8946 in homozygosis or from a donor carrying the wt sequence. PCR products were digested with the suitable restriction enzymes and cloned into the pMIR-REPORT luciferase miRNA expression reporter vector (Ambion) previously digested with *Hind*III and *Sac*I. All plasmids were fully sequenced before use. The dual-luciferase method used to assay miRNA binding affinity for the target sequence was the same used by da Costa Martins *et al.*^41^

COS-7 or HCMa cells were seeded 5,000 cells/well (96-well plate) and transfected with pre-miR-371a-5p, (0.1, 0.5 and 1 nmol/l) or negative control#1 miR precursor molecules, by using Oligofectamine (Invitrogen). Six hours later, cells were co-transfected by using Fugene reagent (Roche) with pMIR-report plasmid (0.15 *μ*g/well) containing BAG3 3′-UTR (wt or carrying the homozygous SNP) and pRL *Renilla* luciferase control reporter vector (10 ng/well), which provides constitutive expression of *Renilla* luciferase and was used for normalization. Firefly luciferase activity was measured 24 h after transfection with a Dual-Glo Luciferase Reporter Assay kit (Promega, Madison, WI, USA) using *Renilla* luciferase as internal control.

### Antibodies and western blotting

Control and treated cells were collected, washed in ice-cold PBS and re-suspended in ice-cold cell disruption buffer (300 *μ*l). miRVana Paris kit (Ambion) and proteins (10 *μ*g) were used for western blot (WB) analysis; blots were probed with anti-BAG3 TOS-2 polyclonal antibody (Biouniversa srl, Fisciano (SA), Italy), anti-phospho-p44/42 MAPK (ERK1/2) antibody (Cell Signaling Technology Inc., Danvers, MA, USA, #9101), anti-p44 MAP kinase ERK1 antibody (Cell Signaling Technology Inc., #4372) or anti ERK2 (C-14) antibody (SC-154, Santa Cruz Biotechnology, Santa Cruz, CA, USA), anti-*β*-catenin antibody (ab32572, Ambion), anti-GAPDH mouse antibody (sc-47724, Santa Cruz Biotechnology) or anti-*β*-actin mouse mAb (sc-47778, Santa Cruz Biotechnology). Nuclear and cytoplasmic fractions were prepared from cells using NE-PER Reagent (Thermo Scientific, Rockford, IL, USA). Nuclear fractions were used for WB analysis with anti-*β*-catenin antibody (ab32572 Ambion), anti-lamin A/C antibody (Cell Signaling Technology Inc.) and anti-GAPDH mouse antibody (sc-47724, Santa Cruz Biotechnology). Immunoreactivity was detected by sequential incubation with horseradish peroxidase-conjugated secondary antibodies (Sigma Aldrich) and ECL detection reagent (GE Healthcare, Amersham Place, Little Chalfont, UK). Signal detection and scanning densitometry of the bands was performed with ImageQuant LAS4000 and ImageQuantTL respectively (GE Healthcare). The results of optical density ratio of target proteins versus either GAPDH, *β*-actin or lamin A are presented as the mean±S.D. of at least three different experiments; each experiment run at least on two different gels.

### Analysis of cell viability

Cells were incubated for the established times in the absence and in the presence of different concentrations of epi. The number of viable cells was quantified by MTT ([3-(4,5-dimethylthiazol-2-yl)-2,5-diphenyl tetrazolium bromide]) assay. Absorption at 550 nm for each well was assessed using a microplate reader (LabSystems, Vienna, VA, USA).

### Apoptosis evaluation

Epi-treated and control cells were collected and then incubated with a propidium iodide (PI) solution (0.1% sodium citrate, 0.1% Triton X-100 and 50 mg/ml of PI) for 30 min at 4 °C. Apoptosis was quantified as the proportion of cells with hypodiploid DNA (sub G0–G1 peak) using flow cytometry (FACScan, Becton Dickinson, Franklin Lakes, NJ, USA). Data were normalized as percentage relative to positive control (doxorubicin).

To analyze Caspase-3 activity, cells (2 × 10^4^) were lysed in a buffer containing HEPES 50 mM, DTT 1 mM, EDTA 0.1 mM, NP-40 0.1% and CHAPS 0.1%, and protein concentration was determined. Protein aliquots (20 *μ*g) were incubated with 20 *μ*M peptide substrate Ac-DEVD-AMC (Pharmingen, San Diego, CA, USA) at 37 °C for 3 h in the lysis buffer. Caspase-3 activity was determined in the cytosolic extracts by analyzing the release of 7-amino-4-methylcoumarin (AMC) monitored by a spectrofluorometer, with excitation wavelength of 380 nm and emission wavelength of 440 nm.

### Analysis of intracellular cAMP

cAMP intracellular concentrations were assayed in HCM cells after epi stimulation. HCMa cells (1 × 10^5^) were seeded in 12-well plates and stimulated with epi at different concentrations (0, 0.5, 5, 50 and 500 *μ*M) for 15 min. Cells were lysed with 300 *μ*l of 0.1 M HCl. Lysates were immediately used for a competitive immunoassay (Direct cyclic AMP Enzyme-linked Immunosorbent Assay kit, Enzo Life Sciences, Farmingdale, NY, USA), to measure the intracellular concentration of cAMP. Data were analyzed by using Microplate Manager 4.0 software (Bio-Rad, Hercules, CA, USA).

### mRNA/miRNA isolation and real-time RT-PCR

Total RNA isolation (mRNA/miRNA) was performed by using the Ambion mirVana PARIS Kit (Ambion). RNA (1 *μ*g) was reverse transcribed by using miScriptII Reverse Transcription Kit (Qiagen) and the HiFlex Buffer. Primers were designed to detect transcripts for hBAG3 (NM_004281.3) and hGAPDH (NM_001256799.1). Real-time PCR was performed on Light Cycler480 (Roche) using LightCycler480 SYBR Green Master Mix. For real-time PCR detection of miRNAs, miScript Primer Assays for miR-371a-5p and RNU6, and the miScript SYBR Green PCR Kit (Qiagen) were used. Transcript quantities were compared using the relative Ct method, where the amount of target normalized to the amount of endogenous control (GAPDH or RNU6) and relative to the control sample is given by 2^(–ΔΔCt)^. The results are presented as mean S.D. of the mean of experimental triplicates.

### Immunofluorescence

In the first experiment aimed at showing *β*-catenin nuclear translocation, HCMa were seeded 100 000 cells/well (6-well plate), and 1 day later were pretreated with the pERK inhibitor U0126 and/or stimulated with epi 500 *μ*mol/l for 15 min. In the second experiment aimed at testing functional effects of BAG3 misregulation, as an *in-vitro* model of TTC, HCM cells were seeded 60 000 cells/well (6-well plates) and transfected with shBAG3-human retroviral construct or HuSH non-effective expression (No target). Seventy-two hours later, cells were treated with epi 500 *μ*mol/l for 2 h. Cells were fixed in 3.7% formaldehyde 1 × PBS for 30 min at room temperature, incubated for 5 min with 1 × PBS 0.1 mol/l glycine, then permealized with 0.1% Triton X-100 and incubated with 5% NGS. Following overnight incubation at 4 °C with rabbit anti-*β*-catenin mAb (1 : 250) (ab32572, Ambion), or mouse anti-BAG-3 mAb (AC-1) (Biouniversa srl, 3 ng/*μ*l), coverslips were washed and incubated at room temperature for 45 min with goat anti-rabbit IgG or anti-mouse IgG DyLigth 488-conjugated antibodies (Jackson ImmunoResearch, West Grove, PA, USA) and then washed, and to stain F-actin filaments cells were incubated 40 min at room temperature with TRITC-conjugated Phalloidin (P1951) (Sigma Aldrich, 1 ng/*μ*l), then cells washed and incubated with Hoechst 33342 (Sigma Aldrich, 2 *μ*g/ml) at room temperature for 10 min. Samples were analyzed using a confocal laser scanning microscope (Zeiss LSM510 Meta Confocal Microscope, Carl Zeiss, Jena, Germany). Images were acquired in sequential scan mode by using the same acquisition parameters. LeicaQ9 Confocal software (Leica Microsystems, Wetzlar, Germany), was used for data analysis.

### Statistical analysis

The results are presented as mean±S.D. Statistical analyses were performed using Microsoft Excel (Microsoft, Redmond, WA, USA) and GraphPad software (GraphPad, San Diego, CA, USA). Student's *t*-test was used to compare two experimental groups and differences were considered significant when *P*<0.05. Allele frequencies were estimated by direct counting. The significance of the distribution of alleles between the patients and the controls was tested by the *χ*^2^-method with Fisher's exact probability test (*P*-value test). The odds ratio of the risk of stress cardiomyopathy was calculated by the 2 × 2 contingency table.

## Figures and Tables

**Table 1:** Summary of the *BAG3* gene mutations identified in TTC patients or HD

**Alleles**	**aa**	**TTC (*****N***)	**%TTC**	**HD (*****N***)	**%HD**
*Homozygous mutations*					
g2252c	3′-UTR	9		6	
c1526t	P407L	2		1	
t757c	C151R	4		3	
Subtotal (A)		15	21.4	10	12.3

*Double heterozygous mutations*					
g2252c and c1526t	3′-UTR P407L	4		7	
g2252c and t757c	3′-UTR C151R	13		7	
g2252c and g1546a	3′-UTR E414K	1		0	
Subtotal (B)		18	25.7	14	17.3

*Single heterozygous mutations*					
g518a	R71Q	3		0	
g2252c	3′-UTR	13		14	
c1526t	P407L	0		0	
g1963a	E553K	1		0	
t757c	C151R	1		0	
Subtotal (C)		18	24.7	14	17.3
					
*Wild type*		19		43	
Subtotal (D)		19	27.1	43	53.1
					
Total		70	100	81	100

Abbreviations: aa, aminoacids; BAG3, BCL2-associated athanogene 3; C, cysteine; E, glutamate; HD (*N*), number of healthy donors; K, lysine; L, leucine; P, proline; Q, glutamine; R, arginine; TTC (*N*), number of Takotsubo patients; UTR, untranslated region

## Abbreviations

BAG3: BCL2-associated athanogene 3
TTC: Takotsubo cardiomyopathy
HCMa: human cardiomyocytes adult
SNP: single-nucleotide polymorphism
miR-371a-5p: microRNA 371a-5p
RT-PCR: reverse-transcription PCR
3′-UTR: 3′-untranslated region
epi: epinephrine
RISC: RNA-induced silencing complex
Ago2: argonaute RISC catalytic component 2
ECG: electrocardiogram
LV: left ventricular
gDNA: genomic DNA
WB: western blotting
RIP: RNA-binding protein immunoprecipitation
cAMP: cyclic AMP
SCR: scrambled
