# Supplementary material for: A novel miR-371a-5p-mediated pathway, leading to BAG3 upregulation in cardiomyocytes in response to epinephrine, is lost in Takotsubo cardiomyopathy
Source: Cell Death Dis. 2015 Oct 29;6(10):e1948–. doi: 10.1038/cddis.2015.280 (PMC4632305; doi:10.1038/cddis.2015.280)
Supplement: Supplementary Information [file cddis2015280x1.docx]

Supplementary data.

**“A novel mir-371-5p-mediated pathway, leading to BAG3 up-regulation in cardiomyocytes in response to epinephrine, is lost in Takotsubo cardiomyopathy”.**

| **Supplementary Table S1. Screening of *BAG3* gene mutations in 70 Takotzubo patients.** | | | | | | | | | | |
| --- | --- | --- | --- | --- | --- | --- | --- | --- | --- | --- |
| samples | age | 3' UTR | 3' UTR | R71Q (ex2) | c151R (ex2) | T155A (ex2) | P407L (ex4) | E414K (ex4) | P545R (ex4) | E553K (ex4) |
| TTC patients |  | g2252c | t2225c | g518a | t757c | g769a | c1526t | g1546a | c1941g | g1963a |
| 1 | 65 |  |  |  |  |  |  |  |  |  |
| 2 | 43 |  |  |  |  |  |  |  |  |  |
| 3 | 81 |  |  | +/- |  |  |  |  |  |  |
| 4 | 35 | -/- |  |  |  |  | +/- |  |  |  |
| 5 | 70 | +/- |  |  |  |  |  |  |  |  |
| 6 | 75 | +/- |  |  |  |  |  |  |  |  |
| 7 | 58 |  |  |  |  |  |  |  |  |  |
| 8 | 60 | +/- |  |  |  |  | +/- |  |  |  |
| 9 | 48 | -/- |  |  | +/- |  |  |  |  |  |
| 10 | 71 | +/- |  |  |  |  | +/- |  |  |  |
| 11 | 55 | +/- |  |  | +/- |  |  |  |  |  |
| 12 | 49 | +/- |  |  | +/- |  |  |  |  |  |
| 13 | 81 |  |  |  | +/- |  |  |  |  |  |
| 14 | 63 |  |  |  |  |  |  |  |  |  |
| 15 | 57 |  |  |  |  |  |  |  |  |  |
| 16 | 63 |  |  |  |  |  |  |  |  |  |
| 17 | 82 | +/- |  |  | +/- |  |  |  |  |  |
| 18 | 61 |  |  |  |  |  |  |  |  |  |
| 19 | 68 | -/- |  |  | +/- |  |  |  |  | +/- |
| 20 | 47 |  |  |  |  |  |  |  |  | +/- |
| 21 | 54 |  |  |  | -/- |  |  |  |  |  |
| 22 | 61 |  |  |  |  |  |  |  |  |  |
| 23 | 62 | +/- |  |  | +/- |  |  |  |  |  |
| 24 | 74 |  |  |  |  |  |  |  |  |  |
| 25 | 69 |  | -/- |  | -/- |  |  |  |  |  |
| 26 | 66 | +/- |  |  |  |  | -/- |  |  |  |
| 27 | 64 | +/- |  |  |  |  | -/- |  |  |  |
| 28 | 45 |  |  | +/- |  |  |  |  |  |  |
| 29 | 50 | +/- |  |  |  |  | +/- |  |  |  |
| 30 | 69 | -/- |  |  |  |  | -/- |  |  |  |
| 31 | 41 | +/- |  |  |  |  |  |  |  |  |
| 32 | 52 | +/- |  |  | -/- |  |  |  |  |  |
| 33 | 70 | -/- |  |  |  |  | +/- |  |  |  |
| 34 | 62 | +/- |  |  |  |  |  |  |  |  |
| 35 | 57 | +/- |  |  |  |  |  |  |  |  |
| 36 | 70 | +/- |  |  |  |  |  |  |  |  |
| 37 | 55 |  |  |  |  |  |  |  |  |  |
| 38 | 72 | -/- |  |  | -/- |  | -/- |  |  |  |
| 39 | 76 | -/- |  |  | -/- |  |  |  |  |  |
| 40 | 72 | +/- |  |  |  |  |  |  |  |  |
| 41 | 70 | +/- |  |  | +/- |  |  |  |  |  |
| 42 | 72 |  |  |  |  |  |  |  |  |  |
| 43 | 81 |  |  | +/- |  |  |  |  |  |  |
| 44 | 65 | +/- |  |  | +/- |  |  |  |  |  |
| 45 | 40 | -/- |  |  | +/- |  | +/- |  |  |  |
| 46 | 70 | +/- |  |  |  |  |  |  |  |  |
| 47 | 57 | +/- |  |  |  |  |  | +/- |  |  |
| 48 | 49 |  |  |  |  |  |  |  |  |  |
| 49 | 81 |  |  |  |  |  |  |  |  |  |
| 50 | 73 | +/- |  |  | +/- |  |  |  |  |  |
| 51 | 77 |  |  |  |  |  |  |  |  |  |
| 52 | 83 | +/- |  |  | +/- |  |  |  |  |  |
| 53 | 66 | +/- |  |  |  |  |  |  |  |  |
| 54 | 81 |  |  |  |  |  |  |  |  |  |
| 55 | 46 | +/- |  |  |  |  | +/- |  |  |  |
| 56 | 70 | +/- |  |  |  |  |  |  |  |  |
| 57 | 57 | +/- |  |  | -/- |  |  |  | +/- |  |
| 58 | 81 |  |  |  |  |  |  |  |  |  |
| 59 | 72 | +/- |  |  | +/- | +/- |  |  |  |  |
| 60 | 61 | -/- |  |  | +/- |  | +/- |  |  |  |
| 61 | 77 | +/- |  |  |  |  |  |  |  |  |
| 62 | 71 |  |  |  |  |  |  |  |  |  |
| 63 | 71 | +/- |  |  |  |  |  |  |  |  |
| 64 | 79 | +/- |  |  | +/- |  |  |  |  |  |
| 65 | 58 | +/- |  |  | +/- |  |  |  |  |  |
| 66 | 80 |  |  |  |  |  |  |  |  |  |
| 67 | 62 | +/- |  |  | +/- |  |  |  |  |  |
| 68 | 73 |  |  |  |  |  |  |  |  |  |
| 69 | 60 | +/- |  |  |  |  |  |  |  |  |
| 70 | 81 | +/- |  |  | +/- |  |  |  |  |  |

| **Supplementary Table S2. Screening of *BAG3* gene mutations in 81 female healthy donors.** | | | | | | | | | | | | | | |
| --- | --- | --- | --- | --- | --- | --- | --- | --- | --- | --- | --- | --- | --- | --- |
| samples | age | 3' UTR | | 3' UTR | R71Q (ex2) | | c151R (ex2) | T155A (ex2) | | P407L (ex4) | E414K (ex4) | | P545R (ex4) | E553K (ex4) |
| DONORS |  | g2252c | | t2225c | g518a | | t757c | g769a | | c1526t | g1546a | | c1941g | g1963a |
| 1 | 50-65 |  | |  |  | |  |  | |  |  | |  |  |
| 2 | 50-65 |  | |  |  | |  |  | |  |  | |  |  |
| 3 | 50-65 |  | |  |  | |  |  | |  |  | |  |  |
| 4 | 50-65 |  | |  |  | |  |  | |  |  | |  |  |
| 5 | 50-65 |  | |  |  | | -/- |  | |  |  | |  |  |
| 6 | 50-65 | +/- | |  |  | | +/- |  | |  |  | |  |  |
| 7 | 50-65 |  | |  |  | |  |  | |  |  | |  |  |
| 8 | 50-65 |  | |  |  | |  |  | |  |  | |  |  |
| 9 | 50-65 |  | |  |  | |  |  | |  |  | |  |  |
| 10 | 50-65 | +/- | |  |  | |  |  | | +/- |  | |  |  |
| 11 | 50-65 |  | |  |  | |  |  | |  |  | |  |  |
| 12 | 50-65 | +/- | |  |  | |  |  | |  |  | |  |  |
| 13 | 50-65 |  | |  |  | |  |  | |  |  | |  |  |
| 14 | 50-65 |  | |  |  | |  |  | |  |  | |  |  |
| 15 | 50-65 | +/- | |  |  | |  |  | |  |  | |  |  |
| 16 | 50-65 |  | |  |  | |  |  | |  |  | |  |  |
| 17 | 50-65 |  | |  |  | |  |  | |  |  | |  |  |
| 18 | 50-65 | +/- | |  |  | |  |  | |  |  | |  |  |
| 19 | 58 |  | |  |  | |  |  | |  |  | |  |  |
| 20 | 55 | +/- | |  |  | | +/- |  | |  |  | |  |  |
| 21 | 60 |  | |  |  | |  |  | |  |  | |  |  |
| 22 | 56 |  | |  |  | |  |  | |  |  | |  |  |
| 23 | 61 | +/- | |  |  | |  |  | | +/- |  | |  |  |
| 24 | 61 | +/- | |  |  | |  |  | | +/- |  | |  |  |
| 25 | 56 |  | |  |  | |  |  | |  |  | |  |  |
| 26 | 61 |  | |  |  | |  |  | |  |  | |  |  |
| 27 | 65 | +/- | |  |  | | -/- |  | |  |  | |  |  |
| 28 | 54 | -/- | |  |  | | -/- |  | |  |  | |  |  |
| 29 | 60 |  | |  |  | |  |  | |  |  | |  |  |
| 30 | 62 | +/- | |  |  | | -/- |  | |  |  | |  |  |
| 31 | 50 | +/- | |  |  | |  |  | | +/- |  | |  |  |
| 32 | 51 | +/- | |  |  | | +/- |  | |  |  | |  |  |
| 33 | 58 |  | |  |  | |  |  | |  |  | |  |  |
| 34 | 50 | -/- | |  |  | | +/- |  | |  |  | |  |  |
| 35 | 58 |  | |  |  | |  |  | |  |  | |  |  |
| 36 | 50 |  | |  |  | |  |  | |  |  | |  |  |
| 37 | 50 |  | |  |  | |  |  | |  |  | |  |  |
| 38 | 50 |  | |  |  | |  |  | |  |  | |  |  |
| 39 | 56 | +/- | |  |  | |  |  | | -/- |  | |  |  |
| 40 | 53 | +/- | |  |  | |  |  | |  |  | |  |  |
| 41 | 51 |  | |  |  | |  |  | |  |  | |  |  |
| 42 | 52 |  | |  |  | |  |  | |  |  | |  |  |
| 43 | 58 |  | |  |  | |  |  | | na |  | |  |  |
| 44 | 50 |  | |  |  | |  |  | |  |  | |  |  |
| 45 | 50 |  | |  |  | |  |  | |  |  | |  |  |
| 46 | 58 | +/- | |  |  | |  |  | | +/- |  | |  |  |
| 47 | 58 |  | |  |  | |  |  | |  |  | |  |  |
| 48 | 59 | +/- | |  |  | |  |  | | +/- |  | |  |  |
| 49 | 54 | -/- | |  |  | | -/- |  | |  |  | |  |  |
| 50 | 57 | +/- | |  |  | |  |  | |  |  | |  |  |
| 51 | 53 | +/- | |  |  | |  |  | |  |  | |  |  |
| 52 | 55 | +/- | |  |  | |  |  | |  |  | |  |  |
| 53 | 58 | +/- | |  |  | | +/- |  | |  |  | |  |  |
| 54 | 51 | +/- | |  |  | |  |  | |  |  | |  |  |
| 55 | 51 | -/- | |  |  | |  |  | |  |  | |  |  |
| 56 | 55 |  | |  |  | |  |  | |  |  | |  |  |
| 57 | 56 |  | |  |  | |  |  | |  |  | |  |  |
| 58 | 55 |  | |  |  | |  |  | |  |  | |  |  |
| 59 | 55 | -/- | |  |  | | +/- |  | |  |  | |  |  |
| 60 | 51 | +/- | |  |  | | +/- |  | |  |  | |  |  |
| 61 | 51 |  | |  |  | |  |  | |  |  | |  |  |
| 62 | 54 | -/- | |  |  | | +/- |  | |  |  | |  |  |
| 63 | 57 |  | |  |  | |  |  | |  |  | |  |  |
| 64 | 62 | +/- | |  |  | |  |  | |  |  | |  |  |
| 65 | 52 |  | |  |  | |  |  | |  |  | |  |  |
| 66 | 50 | +/- | |  |  | | +/- |  | |  |  | |  |  |
| 67 | 59 |  | |  |  | |  |  | |  |  | |  |  |
| 68 | 55 |  | |  |  | |  |  | |  |  | |  |  |
| 69 | 55 |  | |  |  | |  |  | |  |  | |  |  |
| 70 | 53 | +/- | |  |  | |  |  | |  |  | |  |  |
| 71 | 55 | +/- | |  |  | |  |  | |  |  | |  |  |
| 72 | 55 |  | |  |  | |  |  | |  |  | |  |  |
| 73 | 50 |  | |  |  | |  |  | |  |  | |  |  |
| 74 | 50 | +/- | |  |  | | +/- |  | |  |  | |  |  |
| 75 | 53 | +/- | |  |  | |  |  | | +/- |  | |  |  |
| 76 | 58 | +/- | |  |  | |  |  | |  |  | |  |  |
| 77 | 53 | +/- | |  |  | |  |  | |  |  | |  |  |
| 78 | 54 |  | |  |  | |  |  | |  |  | |  |  |
| 79 | 56 |  | |  |  | |  |  | |  |  | |  |  |
| 80 | 56 | +/- | |  |  | |  |  | |  |  | |  |  |
| 81 | 56 |  | |  |  | |  |  | |  |  | |  |  |
| **Supplementary Table S3. Allele frequency of the substitution g2252c** | | | | | | | | | | | | | | |
| 2x2 contingency table | | | | | | | | | | | | | | |
| Allele  Phenotype | | | | c | | | G | | | Total  Alleles | | | Total  Samples  (alleles/2) | |
| HD | | | | 43 | | | 119 | | | **162** | | | **81** | |
| TTC | | | | 53 | | | 87 | | | **140** | | | **70** | |
| **Total** | | | | **96** | | | **206** | | | **302** | | | **151** | |
| **Patients and donors carrying each genotype** | | | | | | | | | | | | | | |
| **Alleles** | | | | **TTC (N)** | | | **%TTC** | | | **HD (N)** | | | **%HD** | |
| c/c | | | | 9 | | | 12.86 | | | 6 | | | 7.41 | |
| c/G | | | | 35 | | | 50.00 | | | 31 | | | 38.27 | |
| G/G | | | | 26 | | | 37.14 | | | 44 | | | 54.32 | |
| **Total** | | | | **70** | | | **100** | | | **81** | | | **100** | |
| C minor allele; G major allele; HD (N) number of healthy donors; TTC (N) number of Takotsubo patients. | | | | | | | | | | | | | | |

| **Supplementary Table S4. miRNAs predicted to bind the major (G) or minor(C) allele of BAG3 mRNA g2252c.** | | | | | |
| --- | --- | --- | --- | --- | --- |
| miRNA | SNP | Predicted | mer | sequence | score |
| hsa-miR-377 | **G** | SLCB | 8:1:0 | AU**C**ACACAAAGGCAACUUUUGU | -8,44 |
| hsa-miR-371-5p | **G** | 5/5 | 8:1:0 | ACU**C**AAACUGUGGGGGCACU | -5,09 |
| hsa-miR-342-3p | **G** | SLCB | 8:1:0 | UCU**C**ACACAGAAAUCGCACCCGU | 0,1 |
| hsa-miR-513c | **G** | 3/5 | 8:1:1 | UUCU**C**AAGGAGGUGUCGUUUAU | 0,39 |
| hsa-miR-942 | **G** | SLCB | 8:1:1 | UCUUCU**C**UGUUUUGGCCAUGUG | 0,37 |
| hsa-miR-617 | **G** | SLCB | 8:1:1 | AGACUUCC**C**AUUUGAAGGUGGC | -3,89 |
| hsa-miR-21 | **G** | SLCB | 8:1:1 | UAGCUUAU**C**AGACUGAUGUUGA | -0,49 |
| mir-616-5p | **G** | Zhang et al, 2010 |  | ACU**C**AAAACCCUUCAGUGACUU | - |
| mir-373* | **G** | Zhang et al, 2010 |  | ACU**C**AAAAUGGGGGCGCUUUCC | - |
| hsa-miR-194 | **C** | SLCB | 8:1:1 | U**G**UAACAGCAACUCCAUGUGGA | 1,51 |
| hsa-miR-653 | **C** | SLCB | 8:1:1 | GUGUU**G**AAACAAUCUCUACUG | -2,03 |
| hsa-miR-1267 | **C** | SLCB | 8:1:1 | CCUGUU**G**AAGUGUAAUCCCCA | 4,88 |
| hsa-miR-606 | **C** | SLCB | 8:1:0 | AAACUACU**G**AAAAUCAAAGAU | -1,99 |
| hsa-miR-216b | **C** | SLCB | 8:1:1 | AAAUCUCU**G**CAGGCAAAUGUGA | -1,59 |
| mir-653 | **C** | Zhang et al, 2010 |  | GUGUU**G**AAACAAUCUCUACUG | - |
| mir-494 | **C** | Zhang et al, 2010 |  | U**G**AAACAUACACGGGAAACCUC | - |
| Wild Type (210-227) UGUUGUUUGAGAAGUUU; Mutated (210-227) UGUUGUUUCAGAAGUUU;  X:Y:Z seed size : mismatch : wobble; ddG: < -10 =strong binding; SLCB predicted only by Segal Lab of computational biology; */* predicted by mirWalk and others / total DBs in mirWalk; NNNN SNP affected binding sites. (Zhang X, *et al*. *Carcinogenesis* 2010; **31**:2118-2123). | | | | | |

Supplementary Figure S1.


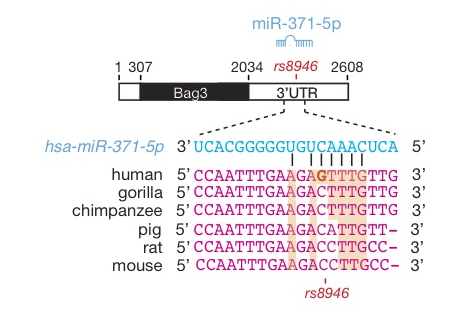


Alignment of BAG3 3' UTR from various species (performed by using mVista tool, Frazer KA et al., 2004), hsa-miR-371a-5p sequence is also shown.

Suplementary Figure S2


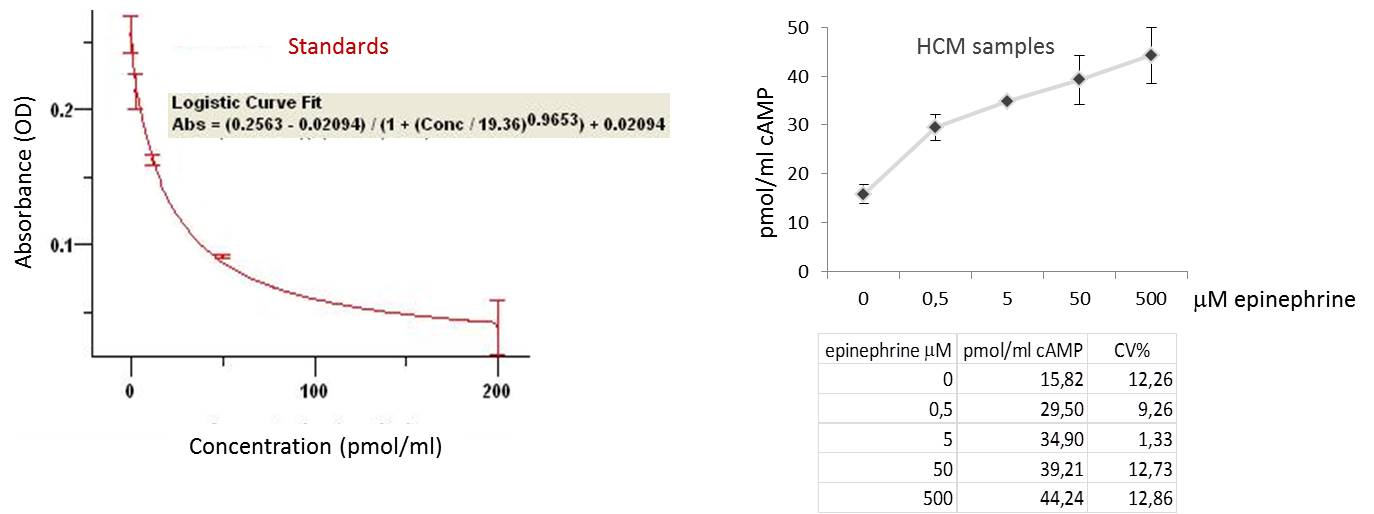


cAMP intracellular concentrations in HCM cells after epinephrine stimulation. 1*10^5 HCM were seeded in 12-well plates and stimulated with epinephrine at different concentrations (0-0.5-5-50-500 μM) for 15 minutes. Cells were lysated with 300 μl of 0.1M HCl. Lysates were immediately used for a competitive immunoassay (Direct cyclic AMP Enzyme-linked Immunosorbent Assay kit, Enzo Life Sciences) to measure the intracellular concentration of cAMP. Data were analyzed by using Microplate Manager 4.0 software.

Supplementary Figure S3


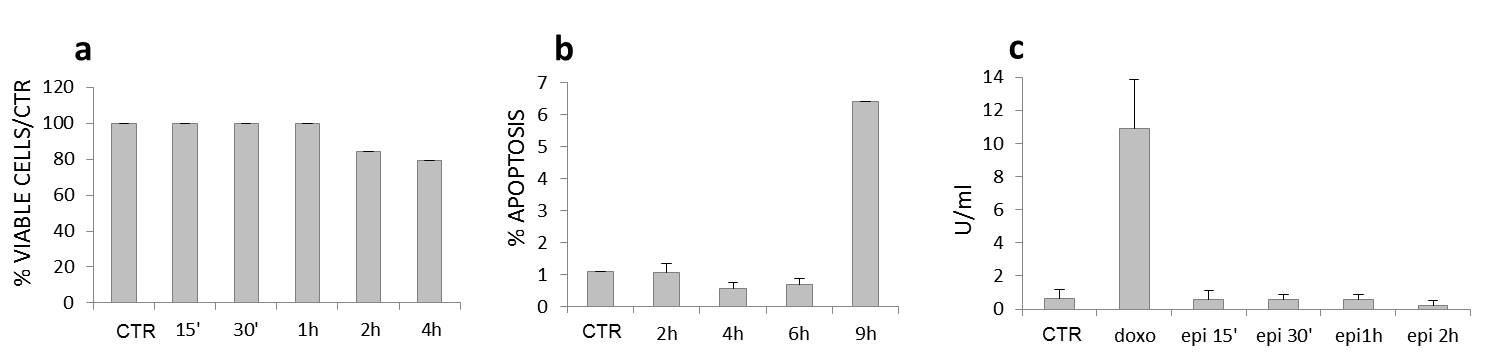


Epinephrine has not effect on cardiomyocytes viability. (A) HCM cells were treated with epinephrine 500 mM for the indicated time points. Cell viability  was evaluated by MTT assay. (B) In the same experimental conditions, apoptosis was evaluated by flow cytometry after permeabilization and propidium iodide staining. (C )Caspase-3 activity was evaluated in cells treated with epinephrine 500 mM for the indicated time points. Doxorubicin 1mM was used as positive control.

Supplementary Figure S4


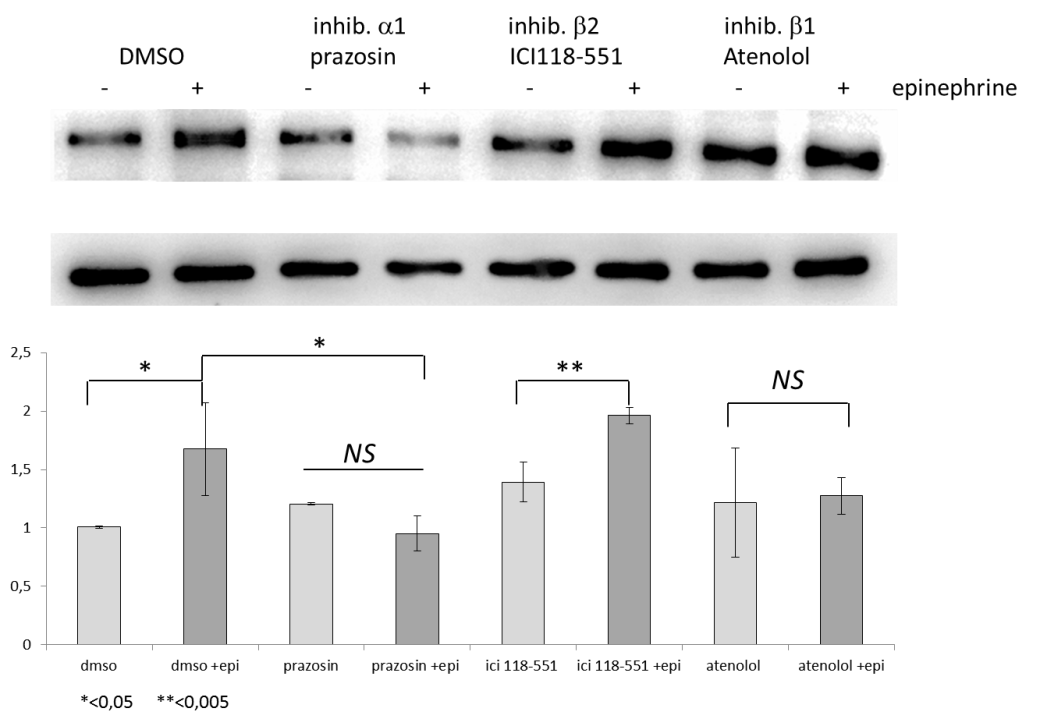


Receptors involved in epinephrine-mediated BAG3 upregulation. 2*10^5 HCMa cells were seeded in 6-well plate and pre-treated for 30 minutes with DMSO alone or alpha 1 antagonist Prazosin (5μM), beta 2 antagonist ICI 118-551 (1μM), beta 1 antagonist Atenolol (10μM). HCMa cells were then stimulated for 2h with epinephrine HCl (500μM) and collected. BAG3 (up) and GAPDH (down) protein levels were analysed by Western blot, one of 3 independent experiments is shown.

Supplementary Figure S5


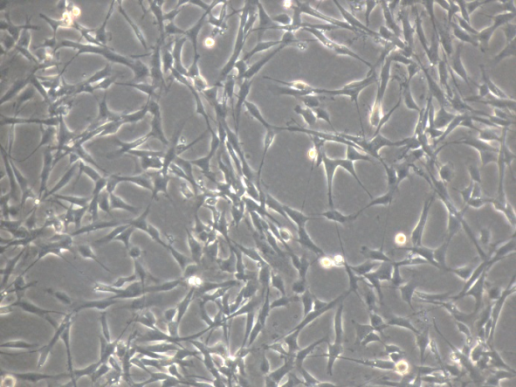


10x magnitude bright field picture of adult Primary Human Cardiac Myocytes (HCM), isolated from the ventricles of the adult heart. The experiments for this paper were carried out with cells purchased both by ScienceCell and PromoCell, qualified for in vitro research on cardiac diseases. Initially, the HCM act more like progenitor cells in that they are not yet fully differentiated. They express the markers of early stage differentiation such as GATA-4 and sarcomeric alpha-actin and have a high capacity for proliferation. When they are grown to confluence and cultivated for an extended period of time, the differentiation process begins. Markers of late differentiation (e.g. sarcomeric alpha-actin, slow muscle myosin) are increased and the cells begin to form myotube-like structures. More details are available on the distributor web site: [www.promocell.com/fileadmin/promocell/PDF/C-12810.pdf](http://www.promocell.com/fileadmin/promocell/PDF/C-12810.pdf)

[www.promocell.com/fileadmin/knowledgebase/pdf-xls/Info_sheet_HCM1.pdf](http://www.promocell.com/fileadmin/knowledgebase/pdf-xls/Info_sheet_HCM1.pdf)
